# An interpretable machine learning model using routine clinical indicators for early pediatric sepsis risk stratification: a multicenter retrospective cohort study

**DOI:** 10.3389/fcimb.2026.1933201

**Published:** 2026-10-28

**Authors:** Yadong Li, Xingcai Li, Dong Wang, Mingjie Li, Wei Pan, Heng Xue

**Affiliations:** 1 Department of Clinical Laboratory, Fujian Children’s Hospital (Fujian Branch of Shanghai Children’s Medical Center), College of Clinical Medicine for Obstetrics & Gynecology and Pediatrics, Fujian Medical University, Fuzhou, China; 2 Department of Anesthesiology, Fujian Medical University Union Hospital, Fuzhou, China; 3 Infectious Disease Department, College of Clinical Medicine for Obstetrics & Gynecology and Pediatrics, Fujian Children’s Hospital (Fujian Branch of Shanghai Children’s Medical Center), Fujian Medical University, Fuzhou, China; 4 Department of Laboratory Medicine, Fujian Medical University Union Hospital, Fuzhou, China; 5 Department of Clinical Laboratory, Children’s Hospital of Nanjing Medical University, Nanjing, China; 6 Department of Clinical Laboratory, Fujian Maternity and Child Health Hospital, College of Clinical Medicine for Obstetrics and Gynecology and Pediatrics, Fujian Medical University, Fuzhou, China

**Keywords:** Gram-stain, machine learning, pediatrics, random forest, sepsis

## Abstract

**Background:**

Sepsis remains a leading cause of pediatric morbidity and mortality worldwide. The 2024 Phoenix Sepsis Criteria emphasize early recognition of organ dysfunction. Conventional diagnostics like blood cultures are time-consuming, and existing machine learning models lack early pathogen-level classification. A machine learning framework for early pediatric sepsis warning and Gram-stain pathogen classification was developed and validated using routine clinical indicators.

**Methods:**

This multicenter retrospective study included 784 children: 432 from Fujian Children’s Hospital for model development and 352 from the Pediatric Intensive Care database of the Children’s Hospital, Zhejiang University School of Medicine for external-cohort evaluation. From 56 clinical variables and 9 derived indices, features were selected in the training cohort by univariable analysis, least absolute shrinkage and selection operator (LASSO) regression, and area under the receiver operating characteristic curve (AUC) evaluation. Eight algorithms were compared, with external-cohort accuracy also informing hyperparameter selection. Shapley additive explanations (SHAP) were used to interpret predictions. The Gram-stain nomogram was developed and evaluated in the pathogen-confirmed training sub-cohort.

**Results:**

The reported models used 12 features. Random forest achieved an AUC of 0.887 (95% confidence interval: 0.850–0.924) and accuracy of 82.1% in the external cohort. Decision curve analysis suggested potential clinical net benefit. C-reactive protein, procalcitonin, alanine aminotransferase, and white blood cell count contributed most to model output. SHAP plots described nonlinear associations within the fitted model. The exploratory Gram-stain nomogram achieved an apparent AUC of 0.753 (95% confidence interval: 0.661–0.844) in the development sub-cohort, with sensitivity of 57.1% and specificity of 80.3% for Gram-positive infection.

**Conclusion:**

The random forest model showed potential for early pediatric sepsis risk stratification and warrants evaluation in an independent cohort. The Gram-stain nomogram provides exploratory findings that require external validation before clinical use. Antibiotic selection and de-escalation should be guided by microbiological evidence and comprehensive clinical assessment.

## 1 Introduction

Sepsis is a major driver of high morbidity and mortality among children globally (Weiss and Fitzgerald, 2024). Despite continued advances in medical care, case-fatality rates for neonatal and pediatric sepsis remain unacceptably high in developed countries. The strong early compensatory capacity of pediatric patients, combined with non-specific clinical manifestations, renders the disease highly prone to rapid deterioration. Sepsis is fundamentally defined as life-threatening organ dysfunction caused by a dysregulated host response to infection (Guo et al., 2023). In 2024, an international consensus group introduced the “Phoenix Sepsis Criteria” based on large-scale cohort data, further emphasizing early recognition of organ dysfunction as a cornerstone of outcome improvement (Schlapbach et al., 2024).

Blood culture is an established method for identifying bloodstream pathogens; however, it is inherently time-consuming and susceptible to interference from bacterial growth characteristics, contamination by non-pathogenic microorganisms, and inappropriate sampling. The resulting false-negative and false-positive rates not only risk delaying targeted therapy but also contribute to unnecessary consumption of medical resources (Li et al., 2025). Consequently, the development of early and rapid diagnostic approaches is critical for outcome optimization.

Early risk stratification represents a pivotal step in reducing sepsis-associated “preventable harm” (Alpern et al., 2025). In recent years, machine learning (ML) has demonstrated promise in predicting pediatric sepsis outcomes; however, most existing models focus on mid-to-late mortality prediction, with fewer tools designed for early-stage alerting (Le et al., 2019; Hou et al., 2020; Chanci et al., 2026). In addition, preliminary pathogen-oriented risk stratification before culture results become available is still challenging. This gap often forces early empirical therapy to rely on broad-spectrum antibiotics, which may increase selection pressure for antimicrobial resistance. Therefore, ML models based on routine, readily accessible clinical indicators may help support early sepsis risk stratification and generate exploratory pathogen-oriented signals, provided that such signals are interpreted cautiously and validated externally.

To address these clinical and methodological gaps, this study developed and evaluated a machine learning model for early pediatric sepsis risk stratification using routinely available clinical indicators. The training cohort was derived from Fujian Children’s Hospital, and a second-center validation cohort from the Children’s Hospital, Zhejiang University School of Medicine was used for model selection and performance assessment. As a secondary exploratory analysis, we also examined whether early clinical indicators could provide preliminary information for Gram-stain classification in pathogen-confirmed sepsis cases. This part of the study was intended to generate preliminary evidence for pathogen-oriented stratification and was not designed as a stand-alone tool for antimicrobial treatment decisions.

## 2 Materials and methods

### 2.1 Study design and population

This multicenter retrospective study used data from two centers for model development and evaluation. Reporting was prepared with reference to the Transparent Reporting of a multivariable prediction model for Individual Prognosis Or Diagnosis plus Artificial Intelligence (TRIPOD+AI) statement (Collins et al., 2024). The workflow and eligibility criteria are presented in **Figure 1**.

**Figure 1:**
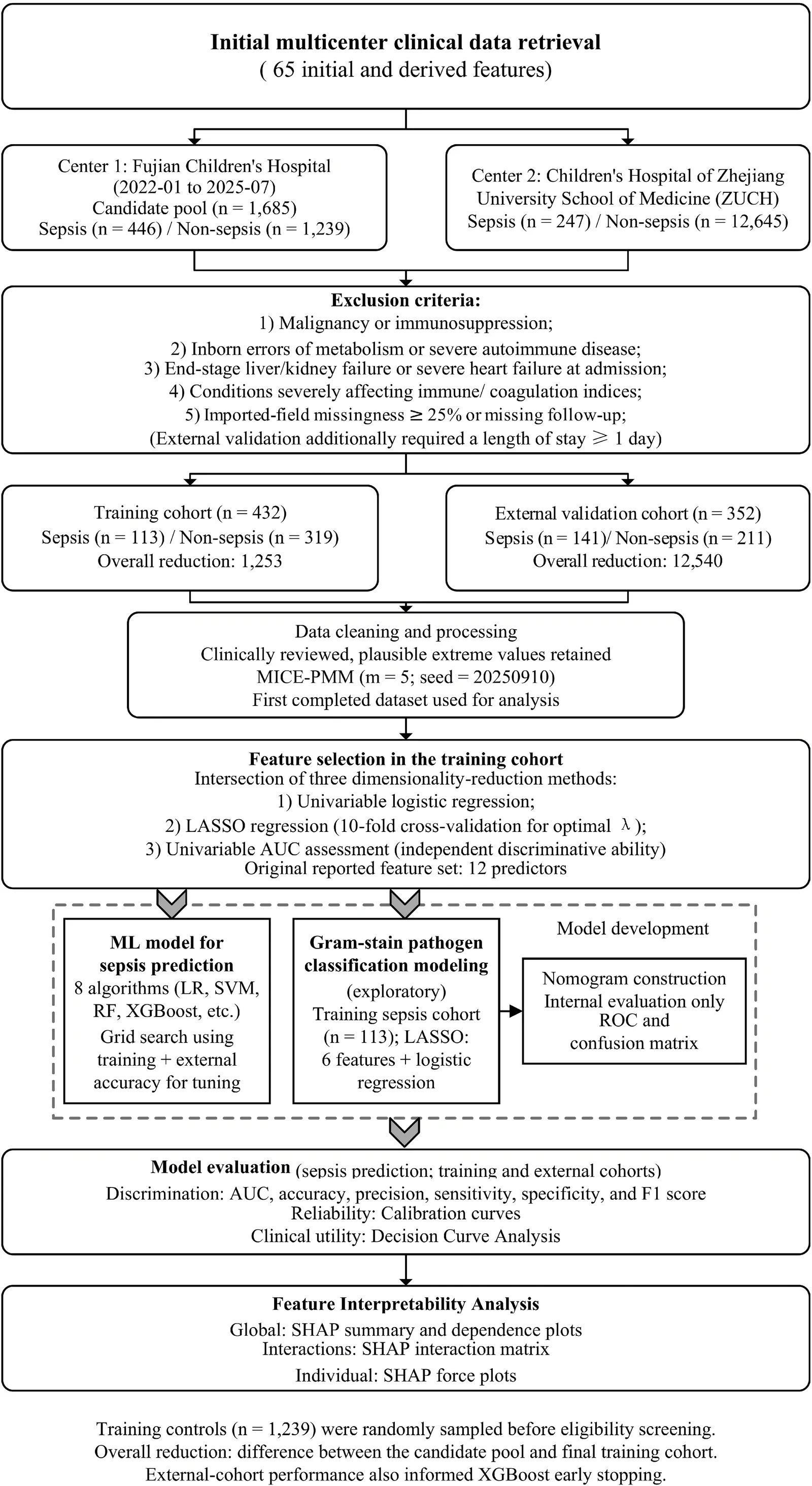
Study workflow and eligibility criteria. Training cohort: 1,685 candidates (446 sepsis, 1,239 non-sepsis) after random sampling of potential controls; 432 included (113 sepsis, 319 non-sepsis). External cohort: 12,892 candidates (247 sepsis, 12,645 non-sepsis); 352 included (141 sepsis, 211 non-sepsis). The length-of-stay criterion of at least one day applied to the external cohort. The model-development feature set comprised 12 predictors, as shown in **Figure 2**. Feature selection used training data; hyperparameter selection also used external-cohort performance.

Training cohort construction: Data were obtained from the electronic health record (EHR) system of Fujian Children’s Hospital for children aged 1 month to 18 years admitted with infection between January 1, 2022, and July 31, 2025. The candidate pool comprised 446 sepsis cases and 1,239 potential non-sepsis controls selected by simple random sampling before eligibility screening, for a total of 1,685 children. After applying the inclusion and exclusion criteria, 432 children were included: 113 with sepsis and 319 with non-septic infections. The overall reduction from the candidate pool to the analysis cohort was 1,253 children. Sepsis was diagnosed independently by at least two senior attending physicians using the 2024 International Consensus Criteria for Pediatric Sepsis and Septic Shock (Phoenix Criteria). Controls had infections such as uncomplicated bacterial pneumonia or acute viral respiratory infections and were assessed clinically and microbiologically as having non-septic infections. Exclusion criteria were: (1) malignancy or immunosuppression; (2) primary genetic or metabolic disorders or severe autoimmune disease; (3) pre-existing end-stage liver or kidney failure or severe chronic cardiac insufficiency; (4) other conditions substantially affecting immune or coagulation indices, including extensive burns, severe polytrauma, or newly diagnosed hematological disorders; and (5) missingness of at least 25% across all fields imported from the corresponding source worksheet or missing follow-up data.

External cohort construction: Intensive care data from the Pediatric Intensive Care (PIC) database of the Children’s Hospital, Zhejiang University School of Medicine (ZUCH) formed a second-center validation cohort used for model selection and performance assessment. The extracted source dataset contained 12,892 children (247 with sepsis and 12,645 without sepsis). The eligibility criteria followed those of the training cohort, with an additional requirement of length of stay (LOS) ≥1 day. Training-cohort enrollment was independent of LOS. The final external cohort included 352 children: 141 with sepsis and 211 with non-septic infections. The overall reduction from the source dataset to the analysis cohort was 12,540 children. The study protocol was approved by the ZUCH Institutional Review Board (Approval No.: 2019_IRB_052). Patient information was anonymized, and the requirement for informed consent was waived for this retrospective study (Guo et al., 2023).

### 2.2 Data collection

A standardized procedure was employed to extract 56 initial clinical variables from EHR systems within 24 hours of patient admission. To comprehensively assess the immunoinflammatory status of the body, 9 derived features were computed and constructed from the initial variables. Collected variables encompassed the following domains:

- Demographic characteristics: Sex and age.
- Complete blood count and differential: White blood cell count (WBC), red blood cell count (RBC), hemoglobin (Hb), mean corpuscular volume (MCV), mean corpuscular hemoglobin (MCH), mean corpuscular hemoglobin concentration (MCHC), hematocrit (HCT), red cell distribution width (RDW), platelet count (PLT), platelet distribution width (PDW), and absolute counts and percentages of neutrophils (NEU), lymphocytes (LYM), monocytes (MONO), eosinophils (EOS), and basophils (BASO).
- Infection and inflammatory markers: C-reactive protein (CRP) and procalcitonin (PCT).
- Biochemical and organ function indices: Potassium (K), sodium (Na), chloride (Cl), calcium (Ca), phosphorus (P), and magnesium (Mg); fasting blood glucose; alanine aminotransferase (ALT), aspartate aminotransferase (AST), gamma-glutamyl transferase (GGT), alkaline phosphatase (ALP), total bilirubin (TBIL), direct bilirubin (DBIL), indirect bilirubin (IBIL), total protein (TP), albumin (ALB), globulin (GLB), and the albumin-to-globulin (A/G) ratio; blood urea nitrogen (BUN), creatinine (CREA), and uric acid (UA); lactate dehydrogenase (LDH), creatine kinase (CK), and its MB isoenzyme (CK-MB); triglycerides (TG) and total cholesterol (CHOL).
- Coagulation function indices: Prothrombin time (PT), international normalized ratio (INR), activated partial thromboplastin time (APTT), thrombin time (TT), fibrinogen (FIB), and D-dimer (D-D).
- Derived immunoinflammatory features: Nine variables were considered. NLR denotes the neutrophil-to-lymphocyte ratio; PLR, the platelet-to-lymphocyte ratio; SII, the systemic immune-inflammation index; and SIRI, the systemic inflammation response index (Ghobadi et al., 2022). CALLY denotes the C-reactive protein-albumin-lymphocyte index (Aoyama et al., 2024). NPAR denotes the neutrophil percentage-to-albumin ratio and was calculated as neutrophil percentage (%) divided by albumin (g/dL) (Li et al., 2024). In the analysis files, the neutrophil proportion (0–1) was multiplied by 100, and albumin (g/L) was divided by 10 before calculating NPAR. The retained analysis labels CLR, dNLR, and MLR represent the lymphocyte-to-C-reactive protein ratio, neutrophil-to-non-lymphocyte ratio, and monocyte-plus-neutrophil-to-lymphocyte ratio, respectively. The other analysis-file formulas were NLR = NEU/LYM; PLR = PLT/LYM; SII = PLT × NEU/LYM; SIRI = NEU × MONO/LYM; CALLY = ALB × LYM/CRP; CLR = LYM/CRP; dNLR = NEU/(WBC − LYM); and MLR = (MONO + NEU)/LYM. Cell counts, ALB, and CRP are reported in 10^9^/L, g/L, and mg/L, respectively. The recorded preprocessing workflow recalculated derived variables after imputation of the source measurements and set infinite values to missing.

### 2.3 Data preprocessing and feature selection

Data cleaning: Obvious data entry errors were removed. Extreme values were reviewed independently by two senior clinicians and retained when considered clinically plausible. Missing values were handled under a missing at random (MAR) assumption using multiple imputation by chained equations (MICE) in R (mice package). Predictive mean matching (PMM) generated five imputed datasets separately within each cohort (m = 5; random seed, 20250910). The imputation inputs comprised 54 laboratory variables in the training cohort and age plus the same 54 laboratory variables in the external cohort. The first completed dataset from each cohort was used for subsequent analysis, and derived indices were recalculated from the completed source measurements.

Feature selection: All 65 candidate predictors entered univariable logistic regression, least absolute shrinkage and selection operator (LASSO) regression, and univariable area under the receiver operating characteristic curve (AUC) evaluation in the training cohort. Predictors were standardized using training-cohort means and standard deviations. Binary LASSO used a penalty selected by 10-fold cross-validation at lambda.min (seed, 20250920). The model-development intersection comprised 12 variables (**Figure 2C**). These criteria combined univariable association, penalized selection, and individual discrimination.

**Figure 2:**
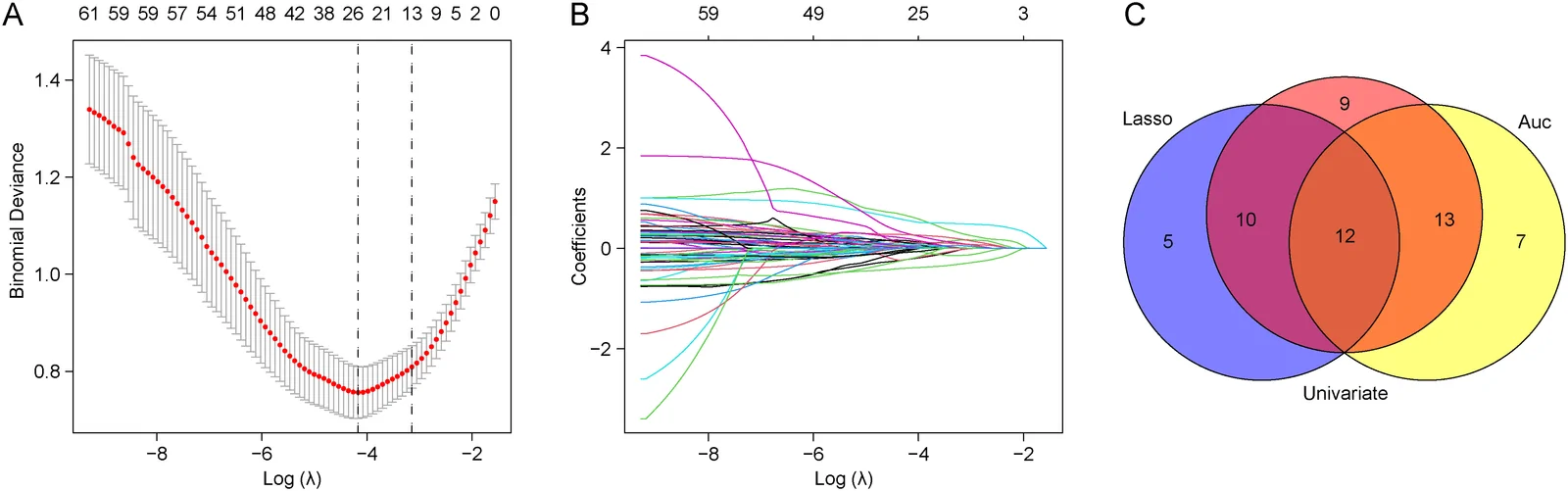
Feature selection in the training cohort. **(A)** Ten-fold cross-validation for LASSO penalty selection. **(B)** LASSO coefficient profiles. **(C)** Intersection of univariable analysis, LASSO selection, and univariable AUC screening. Panels **(A–C)** show the feature-selection results used to develop the 12-predictor models. The NPAR summaries in Section 3.2 and **Supplementary Table 5** use the formula defined in Section 2.2.

### 2.4 Machine learning model construction

Eight ML algorithms were fitted and compared in the training cohort: logistic regression (LR), linear discriminant analysis (LDA), support vector machine (SVM), k-nearest neighbors (KNN), naïve Bayes (NB), decision tree (DT), random forest (RF), and extreme gradient boosting (XGBoost). Hyperparameter settings were compared using grid search. Selection of the retained settings used the sum of training and external-cohort classification accuracy. XGBoost also monitored external-cohort performance during early stopping. Thus, the external cohort contributed to model selection as well as performance assessment.

### 2.5 Model evaluation and interpretability analysis

Model performance was evaluated in the training and external cohorts. Discrimination was assessed using AUC with 95% confidence intervals (CIs). Accuracy, precision, sensitivity, specificity, and F1 score summarized classification performance, with confusion matrices showing the classification counts. Calibration curves compared predicted probabilities with observed event rates. Decision curve analysis (DCA) estimated potential clinical net benefit across threshold probabilities.

### 2.6 Feature interpretability analysis

Shapley additive explanations (SHAP) described feature contributions to RF output. Summary plots ranked features by the magnitude of their contributions, dependence plots showed how contributions varied across feature values, and interaction plots displayed pairwise patterns. Force plots illustrated the combined contributions for individual samples. Interpretation focused on the fitted model and the output scale shown in each plot.

### 2.7 Construction of the Gram-stain classification nomogram

As a secondary exploratory analysis, a model was developed to distinguish Gram-positive from Gram-negative infection in the pathogen-confirmed sepsis sub-cohort of the training set. LASSO regression selected six predictors from the 12 core features, with the penalty parameter chosen by cross-validation. The six predictors were standardized, entered into multivariable logistic regression, and displayed as a nomogram. Receiver operating characteristic (ROC) curves and confusion matrices summarized apparent performance in the same development sub-cohort. Absolute regression coefficients described predictor contributions.

### 2.8 Statistical analysis

Analyses used R version 4.4.3. Within-group distributions were assessed with the Shapiro–Wilk test. Variables with Shapiro–Wilk P > 0.05 in both groups were summarized as mean ± standard deviation (SD) and compared using Welch’s t-tests; other continuous variables were summarized as median [interquartile range (IQR), 25th–75th percentiles] and compared using the Mann–Whitney U test with continuity correction. Categorical variables were reported as counts and percentages and compared using Pearson’s chi-square test or Fisher’s exact test when expected cell counts were below 5. Tests were two-sided, with P < 0.05 indicating statistical significance.

## 3 Results

### 3.1 Study population and baseline characteristics

The final analysis included 784 children. The training cohort included 432 of the 1,685 candidates assessed after random sampling of potential controls, and the external cohort included 352 children from the PIC source dataset of 12,892 children. Sepsis accounted for 26.2% (113/432) of the training cohort and 40.1% (141/352) of the validation cohort. Before feature selection, 56 clinical variables and 9 derived indices were considered. Baseline characteristics are reported in **Supplementary Tables S1, S2**.

Training cohort baseline characteristics: Children with sepsis were younger than those with non-septic infections (median age, 2.0 vs. 4.0 years; P < 0.001). WBC, NEU, and MONO counts were higher in the sepsis group (all P < 0.001), while lymphocyte and eosinophil percentages were lower (P < 0.05). NLR, dNLR, SII, MLR, and SIRI were also higher (P < 0.05), whereas CALLY and CLR were lower (P < 0.001). NPAR was higher in the sepsis group (15.80 ± 5.13 vs. 12.89 ± 4.34; P < 0.001).

External cohort baseline characteristics: Median age was 0.6 [0.2–3.3] years in the sepsis group and 1.5 [0.3–4.8] years in the non-sepsis group (P < 0.05). Both groups were younger than their corresponding training groups. Among children with sepsis, median PCT was 5.29 ng/mL in the validation cohort and 0.59 ng/mL in the training cohort; median TP was 54.1 and 63.0 g/L, respectively. These are descriptive comparisons across cohorts. Within the validation cohort, the P values for sepsis versus non-sepsis comparisons were 0.556 for WBC, 0.597 for NEU, 0.101 for MONO, and 0.957 for dNLR. SII, CALLY, PLR, and CLR were lower in the sepsis group (all P < 0.001; **Supplementary Table 2**). NPAR was higher in the sepsis group (median [Q1–Q3], 20.40 [14.21–27.07] vs. 17.00 [13.23–20.31]; P < 0.001).

Missing-data assessment: Across the 56 source variables, 525 of 24,192 values (2.17%) in the training cohort and 594 of 19,712 values (3.01%) in the external cohort were missing before imputation. Variable-level missingness was highest for D-dimer in the training cohort (16.90%) and magnesium in the external cohort (30.68%). **Supplementary Tables S3, S4** report missing counts and distributions before and after imputation for the laboratory variables and derived indices. The completed datasets preserved all observed values after the recorded unit and coding conversions. The before-and-after comparisons yielded P > 0.05 and serve as descriptive distribution checks. NEU% and ALB were complete in both cohorts, giving identical corrected NPAR values before and after imputation.

### 3.2 Feature selection and dimensionality reduction

Feature selection was performed in the training cohort (n = 432) using univariable association, LASSO regression, and univariable AUC screening. **Figures 2A–C** show the feature-selection results used to develop the 12-predictor models. **Supplementary Table 5** reports univariable AUC summaries using the index definitions in Section 2.2; NPAR was recalculated for these summaries and was not part of the 12-predictor panel.

The 12 predictors used for model development (**Figure 2C**) were grouped as follows:

- Inflammatory and Infectious Markers: WBC, NEU, CRP, PCT, and SIRI
- Erythrocyte-Related Parameters: RBC, HCT, and RDW
- Biochemical Metabolic and Organ Function Indicators: ALT, TBIL, TP, and serum potassium (K)

The ML models used this 12-variable predictor panel.

### 3.3 ML model construction and performance evaluation

The predictive performance and clinical applicability of 8 candidate models were systematically compared using ROC curves, radar charts, and confusion matrices.

Discrimination: RF, XGBoost, SVM, and KNN achieved training AUCs approaching 1.000 (**Figure 3A**). Performance in the external cohort is shown in **Figure 3B**. RF achieved an AUC of 0.887 (95% CI: 0.850–0.924), followed closely by XGBoost at 0.883 (95% CI: 0.845–0.921). Logistic regression achieved an AUC of 0.867, compared with 0.882 in training. The external AUCs for SVM and KNN were 0.702 and 0.727, respectively.

**Figure 3:**
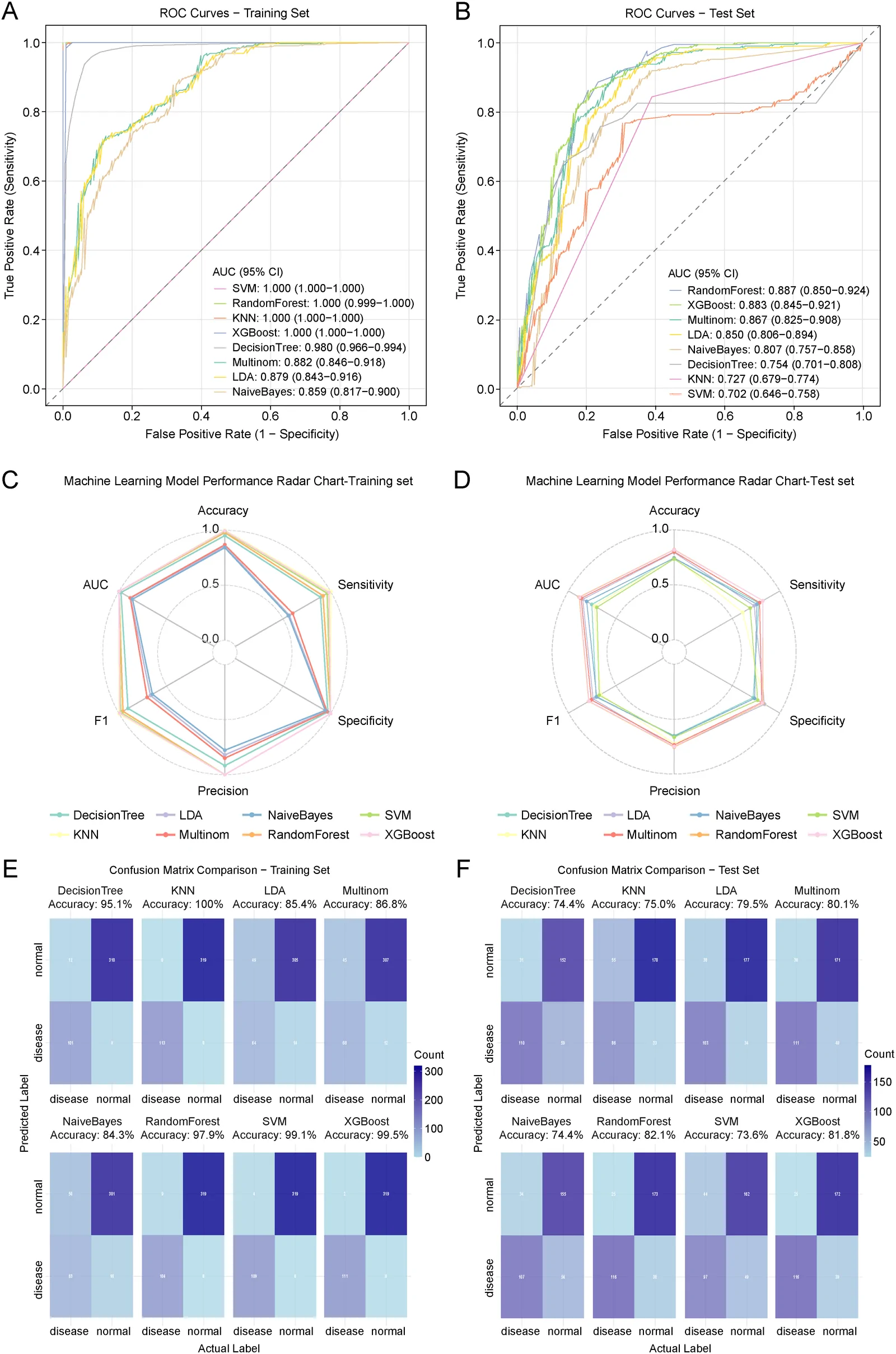
Performance of the eight 12-variable sepsis models. ROC curves in the training **(A)** and external **(B)** cohorts; radar plots of accuracy, AUC, sensitivity, specificity, precision, and F1 score in the training **(C)** and validation **(D)** cohorts; confusion matrices in the training **(E)** and validation **(F)** cohorts.

Classification metrics: The radar plots summarize six performance measures in the training and external cohorts (**Figures 3C, D**). RF achieved an external-cohort accuracy of 82.1% and an F1 score of 0.786, with a similar overall metric profile to XGBoost.

Classification counts: The RF confusion matrix in the external cohort contained 116 true positives, 25 false negatives, 173 true negatives, and 38 false positives, treating sepsis as the positive class (**Figure 3F**). Sensitivity was 82.3% and specificity was 82.0%. RF was selected for further interpretation based on its discrimination, calibration, decision curves, and classification metrics.

### 3.4 Model calibration

Calibration curves compared predicted probabilities with observed sepsis proportions in the training and external cohorts (**Figures 4A, B**). In the external cohort, the RF curve followed the diagonal over part of the probability range and deviated at high predicted probabilities. Calibration across the full range, particularly the high-risk tail, warrants assessment in additional clinical settings (Riley et al., 2024).

**Figure 4:**
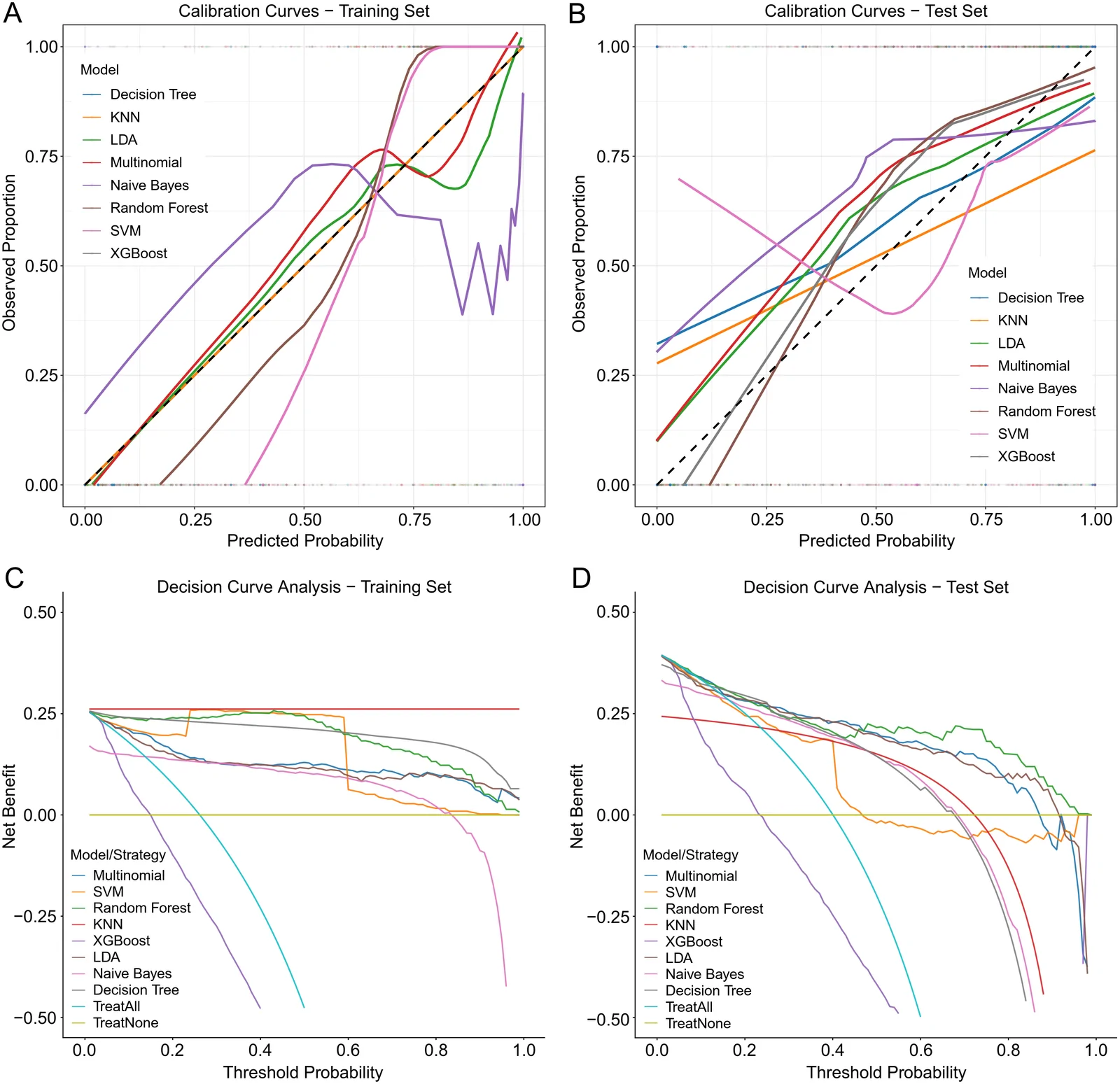
Calibration and decision curve analysis. Calibration plots in the training **(A)** and external **(B)** cohorts; the diagonal indicates perfect agreement. Decision curves in the training **(C)** and validation **(D)** cohorts compare model net benefit with treat-all and treat-none strategies.

### 3.5 Clinical utility assessment (decision curve analysis)

DCA compared the candidate models with treat-all and treat-none strategies in the training and external cohorts (**Figures 4C, D**). RF showed positive net benefit across a range of threshold probabilities in the external cohort. Evaluation of model-guided care would require a prospective study.

### 3.6 Model interpretability and SHAP analysis

SHAP analysis described the contributions of the input variables to the fitted RF model at global and individual levels.

Global feature importance: The SHAP summary plot ranked features by their contributions to RF predictions (**Figure 5A**). CRP, PCT, ALT, and WBC were the four leading contributors. SHAP values describe contributions relative to the model’s reference output.

**Figure 5:**
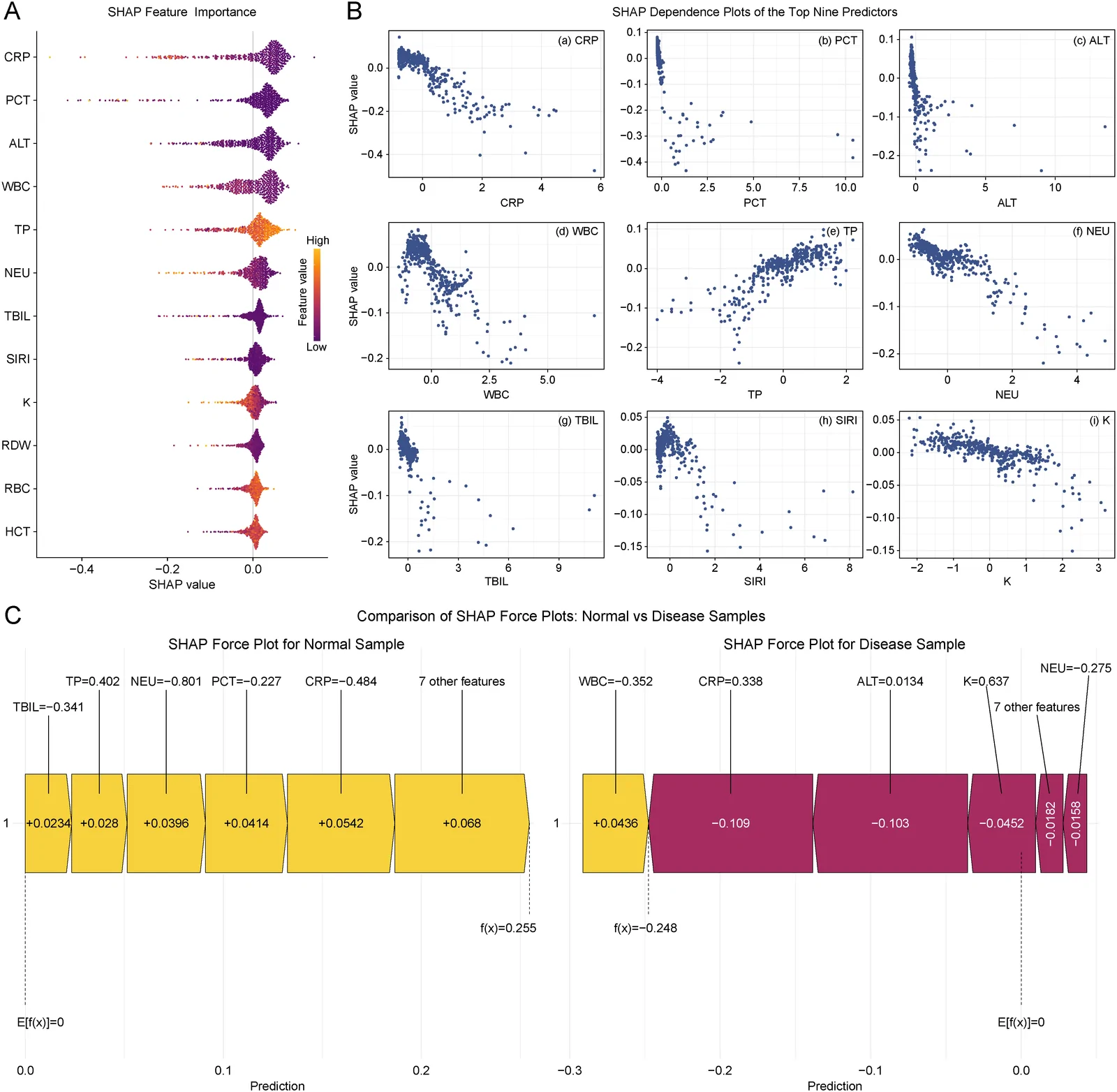
SHAP explanations of the RF model. **(A)** Feature contributions ranked by mean absolute SHAP value. **(B)** Dependence plots for the nine highest-ranked features. **(C)** Force plots for representative non-sepsis and sepsis samples. The axes show contributions on the displayed explanation scale; their interpretation depends on the modeled output class and reference value.

Nonlinear prediction patterns: SHAP dependence plots showed variation in feature contributions across the displayed input values (**Figure 5B**). The patterns for CRP, TP, and the other predictors describe associations within the fitted RF model.

Individual-level explanations: The force plots show how feature contributions accumulate from the displayed reference value for a representative non-sepsis sample and a sepsis sample (**Figure 5C**). Their horizontal coordinates represent the displayed explanation scale.

### 3.7 SHAP interaction analysis

To explore model-level interaction patterns among core clinical features, the SHAP interaction matrix and interaction dependence plots were used to assess whether the RF model incorporated joint information from paired variables (**Figure 6**).

**Figure 6:**
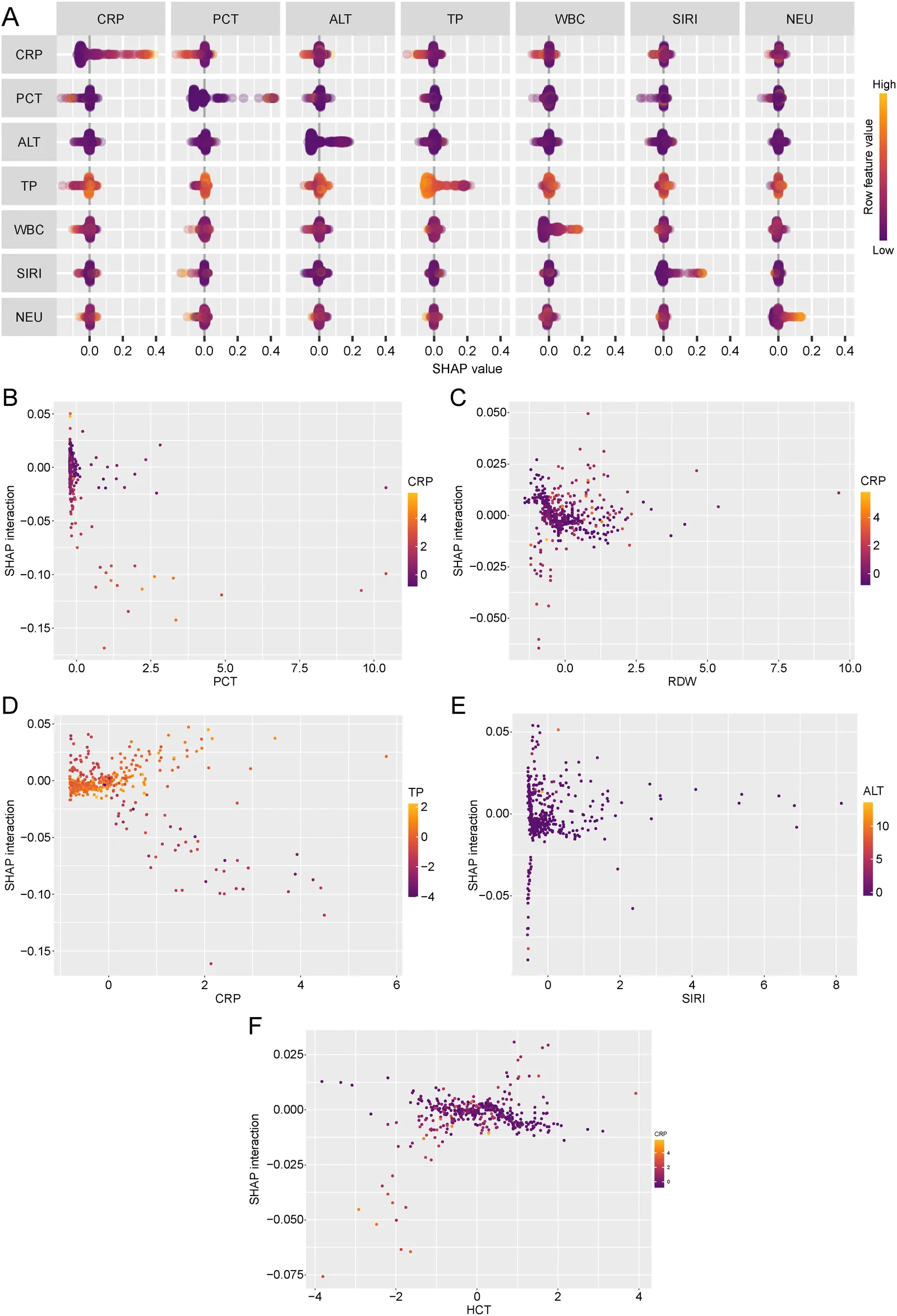
SHAP interaction patterns in the RF model. **(A)** Interaction matrix showing feature main effects on the diagonal and pairwise contributions off the diagonal. Interaction dependence plots for CRP with PCT **(B)**, RDW **(C)**, and TP **(D)**, SIRI with ALT **(E)**, and HCT with CRP **(F)**. These plots describe joint contributions within the fitted model.

Global interaction patterns: The SHAP interaction matrix displayed feature main effects on the diagonal and pairwise contributions off the diagonal (**Figure 6A**). CRP, PCT, and TP showed prominent contributions, including joint patterns involving CRP with PCT and TP.

Bivariate interaction patterns: Interaction dependence plots (**Figures 6B–F**) further illustrated several model-level bivariate patterns:

- CRP with PCT and RDW: The interaction dependence plots showed variation in the joint model contributions of CRP with PCT (**Figure 6B**) and RDW (**Figure 6C**).
- CRP with TP: The joint contribution of CRP and TP varied across their feature values (**Figure 6D**), indicating an interaction pattern within the fitted model.
- Other interaction patterns: Pairwise contributions also varied for SIRI with ALT (**Figure 6E**) and HCT with CRP (**Figure 6F**). These patterns describe the fitted model and suggest relationships for further investigation.

### 3.8 Construction and apparent performance of the Gram-stain classification nomogram

As a secondary exploratory analysis, a classification model distinguishing Gram-positive (G+) from Gram-negative (G-) bacterial infections was constructed within the pathogen-confirmed sepsis sub-cohort of the training set.

Feature selection and modeling: Among the 12 candidate features, LASSO regression optimized by 10- fold cross-validation retained six predictors with non-zero coefficients (**Figures 7A, B**). These predictors were incorporated into a multivariable logistic regression model to construct an exploratory Gram-stain classification nomogram.

**Figure 7:**
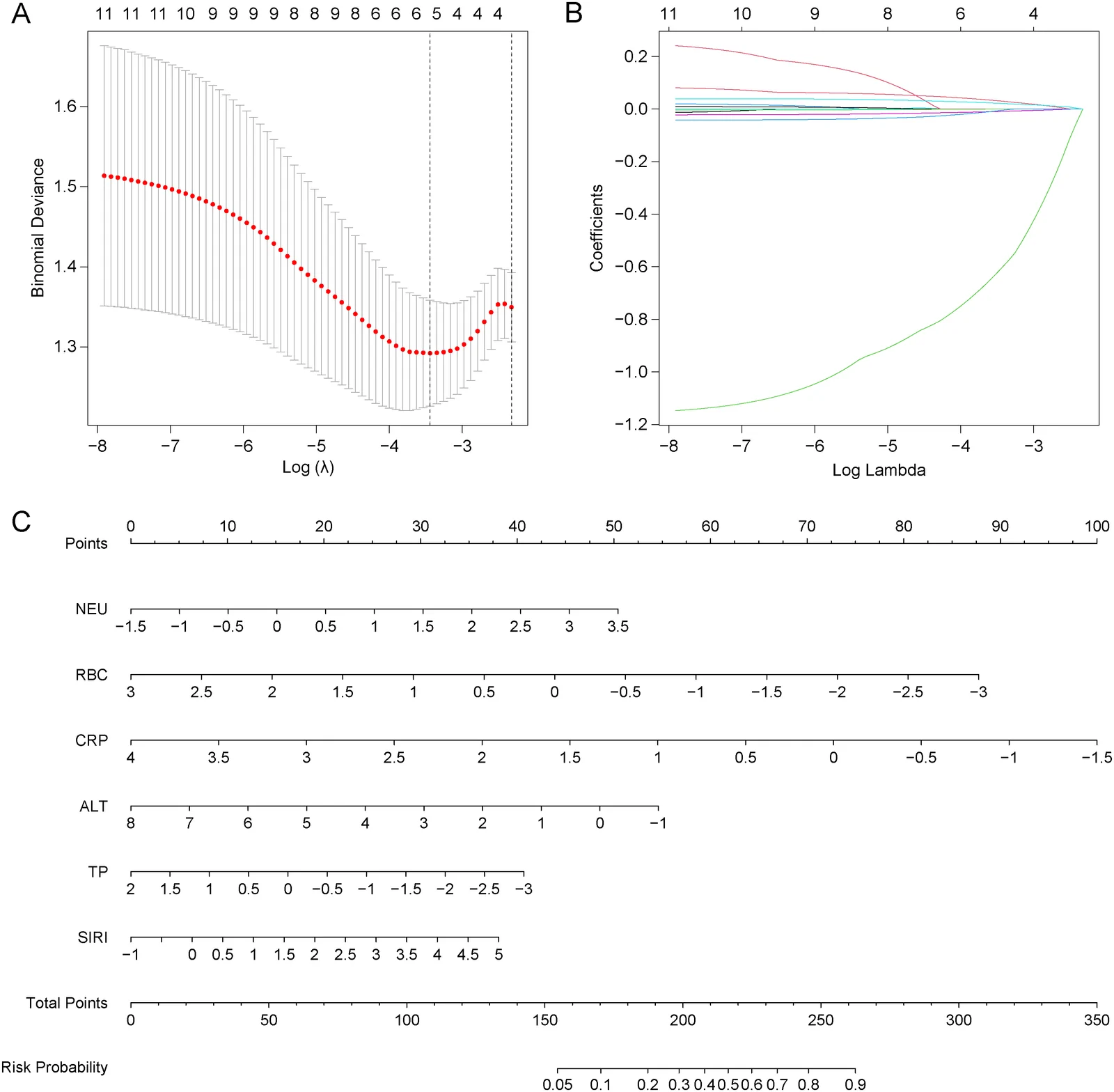
Development of the exploratory Gram-stain nomogram. **(A)** Ten-fold cross-validation for LASSO penalty selection. **(B)** LASSO coefficient profiles. **(C)** Nomogram for Gram-positive infection using six standardized predictors: NEU, RBC, CRP, ALT, TP, and SIRI. Summed points map to the estimated probability. Performance was assessed in the same pathogen-confirmed training sub-cohort.

The coefficient profile suggested that NEU and SIRI were positively associated with the predicted probability of Gram-positive infection, whereas RBC, CRP, ALT, and TP contributed in the opposite direction in this internal model. Based on this logistic regression model, a nomogram was constructed to estimate the probability of Gram-positive infection within the pathogen-confirmed training sub-cohort.

Apparent model performance: The development sub-cohort contained 113 children with sepsis, including 42 with Gram-positive and 71 with Gram-negative infection. The nomogram achieved an AUC of 0.753 (95% CI: 0.661–0.844; **Supplementary Figure S1A**). The predicted-probability distributions overlapped, including around 0.4–0.6 (**Supplementary Figure S1B**). At a probability threshold of 0.44, the confusion matrix yielded an accuracy of 71.7% (81/113). With Gram-positive infection as the positive class, sensitivity was 57.1% (24/42), specificity was 80.3% (57/71), precision was 63.2% (24/38), and the F1 score was 0.600 (**Supplementary Figure S1C**; **Supplementary Table 6**). The exploratory nomogram requires independent external validation before clinical use. Antibiotic selection and de-escalation should be guided by microbiological findings and comprehensive clinical assessment.

## 4 Discussion

This study developed an RF model for early pediatric sepsis risk stratification and evaluated it across two cohorts totaling 784 children. The external-cohort AUC was 0.887, and DCA suggested potential net benefit. Because external-cohort accuracy informed hyperparameter selection, these results describe performance following that selection procedure. The Gram-stain nomogram showed moderate apparent discrimination in the pathogen-confirmed training sub-cohort and remains exploratory.

The cohorts shared several clinical patterns and also differed in age and biomarker distributions. Children with sepsis were younger than their non-sepsis comparators in both cohorts, and the external cohort was younger overall. These findings are consistent with the relevance of age to pediatric sepsis assessment (Carter et al., 2024; Watson et al., 2024).

In the training cohort, higher WBC and NEU counts and lower lymphocyte percentages were accompanied by differences in several derived inflammatory indices. These findings are consistent with clinical associations between immune-cell profiles and pediatric sepsis (Shen et al., 2025). CALLY and CLR were lower in the sepsis group in both cohorts, supporting further investigation of combined inflammatory and nutritional markers (Zhang et al., 2024; Wang et al., 2025).

In the external cohort, comparisons of WBC, NEU, MONO, and dNLR between the sepsis and non-sepsis groups yielded P values of 0.556, 0.597, 0.101, and 0.957, respectively. SII, CALLY, PLR, and CLR were lower in children with sepsis. The younger population, intensive care setting, and higher sepsis proportion provide context for these cohort differences. Pre-admission antibiotics and blood-sampling time within the first 24 hours may also influence the measurements; their contributions require evaluation using treatment and sampling records. Group comparisons and contributions within a multivariable model address different questions.

In the feature construction phase, this study used a dimensionality reduction strategy combining univariable screening, LASSO regularization, and independent AUC evaluation to extract 12 core predictors from 65 initial variables. This pipeline helped reduce multicollinearity and overfitting risk while retaining clinically interpretable indicators (Shen et al., 2024). In clinical terms, these 12 features broadly covered the infection-inflammation-organ dysfunction continuum of pediatric sepsis:

- Immune-inflammatory cascade: WBC, NEU, CRP, PCT, and SIRI captured infection-and inflammation-related information that is clinically relevant to pediatric sepsis (Cakir and Tayman, 2024).
- Microcirculation and endothelial dysfunction: RDW, HCT, and RBC may reflect hematological and physiological changes associated with critical illness, and their inclusion may provide complementary information beyond classic inflammatory markers (Hassan et al., 2025).
- Biochemical measures: ALT, TBIL, TP, and potassium reflect aspects of liver function, protein status, and electrolyte balance. Their clinical interpretation depends on the broader physiological context of sepsis (Beyer et al., 2022).

Consequently, this feature set should be interpreted as a clinically interpretable predictor set selected by data-driven methods rather than as proof of a specific causal pathway.

RF and XGBoost had the highest external-cohort AUCs among the evaluated models, with overlapping confidence intervals. Their ability to represent nonlinear relationships may help explain this pattern. Logistic regression also showed discrimination across cohorts, with AUCs of 0.882 and 0.867. These results support empirical comparison of algorithms in the intended clinical setting (Christodoulou et al., 2019).

Given that discriminative ability alone (AUC) cannot fully translate into clinical utility, calibration accuracy and decision net benefit are important for evaluating potential clinical usefulness (Vickers et al., 2019). This study confirmed that the RF model maintained favorable calibration and decision-curve performance. In the high-pressure environment of pediatric emergency and pediatric intensive care unit (PICU) settings, missed diagnoses may delay timely intervention, whereas false positives may increase unnecessary resource use and antibiotic exposure (Balamuth and Scott, 2023). The RF model achieved a reasonable balance between sensitivity and specificity, and its DCA suggested clinical net benefit across a broad range of threshold probabilities (Vickers et al., 2019). These findings support the potential role of AI-assisted early warning systems as adjunctive tools for pediatric sepsis risk stratification, rather than as standalone treatment-decision systems (Adams et al., 2022).

SHAP analysis was used to improve the interpretability of the RF model, not to establish causal mechanisms. CRP, PCT, ALT, and WBC were the main contributors to model prediction, which is consistent with their clinical relevance in infection, inflammation, and organ dysfunction. Dependence and interaction plots suggested that the contribution of inflammatory markers varied across different biochemical and hematological contexts, including RDW, TP, SIRI, and ALT. These findings indicate that the RF model captured nonlinear and interaction patterns among routinely measured clinical variables. However, these SHAP-derived patterns should be interpreted as model-level associations and hypothesis-generating signals, not as direct evidence of biological mechanisms.

The observed interaction between CRP and RDW may suggest that erythrocyte-related variation modifies the predictive contribution of systemic inflammation in the model. Similarly, the interaction between CRP and TP indicates that the same inflammatory signal may carry different predictive weights under different protein or nutritional backgrounds. The SIRI-ALT interaction and the HCT-CRP interaction further suggest that inflammatory, hepatic, and hematological indicators may jointly contribute to model-based risk estimation. These findings provide interpretable clues for future biological and clinical investigation, but they do not prove specific pathophysiological pathways.

The Gram-stain classification nomogram showed moderate apparent discrimination in the pathogen-confirmed training sub-cohort (AUC = 0.753, 95% CI: 0.661-0.844). Its six selected indicators may reflect host-response patterns associated with different bacterial groups, but the present study cannot establish pathogen-specific mechanisms. Therefore, this nomogram should be viewed as an exploratory risk-stratification model that generates preliminary Gram-stain-oriented information before microbiological confirmation.

In terms of clinical translation, caution is required. When Gram-positive infection was treated as the positive class, the nomogram showed limited sensitivity for Gram-positive identification (57.1%) and a specificity of 80.3%. Accordingly, it should not be used independently to guide antimicrobial selection, antimicrobial de-escalation, or exclusion of Gram-positive infection. The overlap in the intermediate probability range further indicates that cases in this gray zone require standard microbiological testing, local epidemiological knowledge, and comprehensive clinical assessment. External validation in independent pathogen-confirmed cohorts is essential before this model can be considered for clinical implementation.

Potential use of the RF model would involve clinicians assessing children with infection during early hospital care. The R-based research workflow uses 12 indicators collected within 24 hours of admission: WBC, NEU, CRP, PCT, SIRI, RBC, HCT, RDW, ALT, TBIL, TP, and potassium. The intended output is an estimated probability of sepsis to be considered alongside clinical findings. Implementation would require the fixed model, its preprocessing steps and predictor units, checks for implausible inputs, and a validated procedure for missing predictors. Prospective assessment of calibration, decision thresholds, and workflow effects would inform clinical deployment.

### 4.1 Limitations and future work

Several aspects of the study guide further evaluation. The Gram-stain nomogram was developed and assessed in the same pathogen-confirmed sub-cohort, making independent evaluation a priority. The external cohort required a length of stay of at least one day, whereas training-cohort enrollment was independent of length of stay. This difference may introduce selection bias by altering the representation of children discharged, transferred, or dying during the first day. Evaluation under harmonized eligibility criteria would clarify performance in these early presentations. The retrospective design also leaves scope for residual confounding by pre-admission antibiotics and early treatment. Finally, the validation cohort also contributed to hyperparameter selection and XGBoost early stopping; evaluation of the fixed model in a further independent cohort would strengthen the evidence for generalizability (Riley et al., 2024).

The model provides a static assessment from measurements collected within 24 hours of admission. Future studies could combine serial laboratory measurements with clinical observations to develop dynamic early-warning models and compare them with this baseline approach. Prospective work could also assess calibration at high predicted probabilities. Additional microbiological methods, including metagenomic next-generation sequencing (mNGS), may broaden pathogen confirmation for future Gram-stain analyses.

## 5 Conclusions

This study developed an RF model and evaluated it in a second-center cohort based on routinely available early clinical indicators for pediatric sepsis prediction. SHAP analysis improved the transparency of model behavior by identifying the variables most strongly contributing to prediction and by illustrating model-level nonlinear associations. The exploratory Gram-stain classification nomogram showed moderate apparent performance in the pathogen-confirmed training sub-cohort, but it requires independent external validation before clinical application. Overall, this study supports the potential value of interpretable machine learning for early pediatric sepsis risk stratification, while pathogen-oriented prediction and antimicrobial decision support require further prospective validation.

## Data Availability

The raw data supporting the conclusions of this article will be made available by the authors, without undue reservation.
