# Supplementary material for: An interpretable machine learning model using routine clinical indicators for early pediatric sepsis risk stratification: a multicenter retrospective cohort study
Source: Front Cell Infect Microbiol. 2026 Oct 28;16:1933201. doi: 10.3389/fcimb.2026.1933201 (PMC13751490; doi:10.3389/fcimb.2026.1933201)
Supplement: Supplementary file 1 [file DataSheet1.docx]

Supplementary material

Manuscript ID 1933201

Supplementary Figure S1. Apparent performance of the exploratory Gram-stain model in its development sub-cohort. (A) ROC curve. (B) Predicted-probability distributions; the dashed line marks the reference probability of 0.5. (C) Confusion matrix at a classification threshold of 0.44, with 1 denoting Gram-positive and 0 Gram-negative infection. (D) Absolute coefficients for standardized predictors. Gram-positive sensitivity was 57.1%. Treatment decisions should be guided by microbiological evidence and clinical assessment.

# Abbreviations used in Tables S1–S5

WBC, white blood cell count; NEU, neutrophils; LYM, lymphocytes; MONO, monocytes; EOS, eosinophils; BASO, basophils; RBC, red blood cell count; Hb, hemoglobin; MCV, mean corpuscular volume; MCH, mean corpuscular hemoglobin; MCHC, mean corpuscular hemoglobin concentration; HCT, hematocrit; RDW, red cell distribution width; PLT, platelet count; PDW, platelet distribution width; CRP, C-reactive protein; PCT, procalcitonin; K, potassium; Na, sodium; Cl, chloride; Ca, calcium; P, phosphorus; Mg, magnesium; GLU, glucose; ALT, alanine aminotransferase; AST, aspartate aminotransferase; GGT, gamma-glutamyl transferase; ALP, alkaline phosphatase; TBIL, total bilirubin; DBIL, direct bilirubin; IBIL, indirect bilirubin; TP, total protein; ALB, albumin; GLOB, globulin; A/G, albumin-to-globulin ratio; BUN, blood urea nitrogen; CREA, creatinine; UA, uric acid; LDH, lactate dehydrogenase; CK, creatine kinase; CK-MB, creatine kinase-MB; TG, triglycerides; CHOL, total cholesterol; PT, prothrombin time; INR, international normalized ratio; APTT, activated partial thromboplastin time; TT, thrombin time; FIB, fibrinogen; PLR, platelet-to-lymphocyte ratio; CALLY, C-reactive protein-albumin-lymphocyte index; CLR, study label for the lymphocyte-to-C-reactive protein ratio; NLR, neutrophil-to-lymphocyte ratio; dNLR, study label for the neutrophil-to-non-lymphocyte ratio; SII, systemic immune-inflammation index; NPAR, neutrophil percentage-to-albumin ratio; MLR, retained study analysis label for the monocyte-plus-neutrophil-to-lymphocyte ratio; SIRI, systemic inflammation response index.

NPAR was calculated as neutrophil percentage (%) divided by albumin (g/dL). In the analysis files, the neutrophil proportion (0–1) was multiplied by 100, and albumin (g/L) was divided by 10 before calculating NPAR. The source measurements were complete in all 784 participants. Other formulas are described in Section 2.2 of the main text.

## Supplementary Table S1. Clinical baseline characteristics of the sepsis and non-sepsis groups in the training cohort

| **Characteristic** | **Sepsis group (n = 113)** | **Non-sepsis group (n = 319)** | **P value** |
| --- | --- | --- | --- |
| Sex, n (%) | Male, 77 (68.1%) Female, 36 (31.9%) | Male, 175 (54.9%) Female, 144 (45.1%) | 0.014 |
| Age, years | 2.000 [1.000–5.000] | 4.000 [2.000–7.000] | <0.001 |
| WBC, ×10⁹/L | 13.37 [9.15–18.45] | 8.71 [6.22–12.16] | <0.001 |
| Neutrophils (%) | 63.6 [46.9–78.9] | 55.5 [44.6–68.9] | 0.002 |
| Lymphocytes (%) | 27.1 [14.3–43.1] | 34.3 [20.8–44.2] | 0.001 |
| Monocytes (%) | 7.2 [5.6–10.2] | 7.6 [5.6–9.9] | 0.883 |
| Eosinophils (%) | 0.2 [0.1–1.0] | 0.5 [0.1–1.6] | 0.011 |
| Basophils (%) | 0.1 [0.0–0.1] | 0.1 [0.1–0.2] | <0.001 |
| NEU, ×10⁹/L | 7.42 [5.01–13.28] | 4.62 [2.87–7.23] | <0.001 |
| LYM, ×10⁹/L | 2.93 [1.77–5.54] | 2.53 [1.72–4.21] | 0.168 |
| MONO, ×10⁹/L | 0.98 [0.63–1.52] | 0.64 [0.42–0.94] | <0.001 |
| EOS, ×10⁹/L | 0.03 [0.01–0.13] | 0.04 [0.01–0.15] | 0.465 |
| BASO, ×10⁹/L | 0.01 [0.00–0.02] | 0.01 [0.00–0.02] | 0.207 |
| RBC, ×10¹²/L | 4.19 [3.77–4.50] | 4.44 [4.16–4.75] | <0.001 |
| Hb, g/L | 114.0 [103.0–121.0] | 122.0 [115.0–130.5] | <0.001 |
| MCV, fL | 82.4 [78.6–86.9] | 82.1 [78.8–84.9] | 0.190 |
| MCH, pg | 27.8 [26.1–28.9] | 27.6 [26.6–28.7] | 0.692 |
| MCHC, g/L | 334.0 [325.0–343.0] | 337.0 [330.0–344.0] | 0.102 |
| HCT (%) | 33.6 ± 5.1 | 36.4 ± 3.7 | <0.001 |
| RDW (%) | 13.9 [13.0–15.1] | 13.2 [12.7–13.8] | <0.001 |
| PLT, ×10⁹/L | 291 [226–385] | 278 [225–357] | 0.299 |
| PDW (%) | 15.8 [15.5–16.3] | 15.8 [15.5–16.0] | 0.135 |
| CRP, mg/L | 35.8 [15.0–73.7] | 10.0 [3.4–24.0] | <0.001 |
| PCT, ng/mL | 0.59 [0.27–9.70] | 0.16 [0.09–0.37] | <0.001 |
| K, mmol/L | 4.5 ± 0.6 | 4.3 ± 0.5 | <0.001 |
| Na, mmol/L | 136 [133–138] | 137 [135–139] | <0.001 |
| Cl, mmol/L | 102 [99–103] | 101 [99–103] | 0.934 |
| Ca, mmol/L | 2.35 [2.21–2.45] | 2.36 [2.27–2.46] | 0.168 |
| P, mmol/L | 1.48 [1.29–1.70] | 1.48 [1.30–1.65] | 0.651 |
| Mg, mmol/L | 0.91 [0.83–0.97] | 0.92 [0.87–0.97] | 0.132 |
| GLU, mmol/L | 5.6 [5.0–6.4] | 5.5 [5.0–6.4] | 0.945 |
| ALT, U/L | 25.8 [15.2–48.6] | 15.9 [11.4–22.5] | <0.001 |
| AST, U/L | 38.0 [27.2–63.0] | 37.0 [28.2–46.9] | 0.411 |
| GGT, U/L | 16.6 [11.0–55.0] | 11.0 [9.0–15.0] | <0.001 |
| ALP, U/L | 198.0 [159.0–240.0] | 183.7 [149.0–233.7] | 0.088 |
| TBIL, µmol/L | 6.9 [4.3–10.4] | 5.2 [3.8–7.0] | <0.001 |
| DBIL, µmol/L | 2.5 [1.5–4.6] | 1.6 [1.0–2.5] | <0.001 |
| IBIL, µmol/L | 3.9 [2.8–6.3] | 3.6 [2.6–4.9] | 0.066 |
| TP, g/L | 63.00 [57.00–67.60] | 67.40 [64.30–71.00] | <0.001 |
| ALB, g/L | 40.8 [37.0–43.0] | 43.8 [41.5–46.0] | <0.001 |
| GLOB, g/L | 21.6 [18.0–26.0] | 24.0 [20.8–26.9] | <0.001 |
| A/G | 1.90 [1.60–2.30] | 1.80 [1.60–2.10] | 0.260 |
| BUN, mmol/L | 3.5 [2.5–4.4] | 3.5 [2.8–4.4] | 0.635 |
| CREA, µmol/L | 26.0 [21.0–33.0] | 30.0 [24.0–37.0] | <0.001 |
| UA, µmol/L | 218.0 [150.0–261.0] | 248.0 [204.0–305.0] | <0.001 |
| LDH, U/L | 299.0 [240.0–402.0] | 284.0 [238.6–346.7] | 0.115 |
| CK, U/L | 71.8 [45.0–122.0] | 95.5 [67.2–143.3] | <0.001 |
| CK-MB, U/L | 27 [21–40] | 26 [20–35] | 0.285 |
| TG, mmol/L | 1.10 [0.80–1.49] | 0.92 [0.70–1.36] | 0.011 |
| CHOL, mmol/L | 3.39 [2.60–4.12] | 3.70 [3.27–4.20] | <0.001 |
| PT, s | 13.90 [13.00–14.70] | 13.50 [12.90–14.00] | 0.001 |
| INR | 1.07 [0.98–1.14] | 1.03 [0.98–1.09] | 0.018 |
| APTT, s | 38.9 [35.0–43.7] | 38.3 [35.3–41.4] | 0.334 |
| TT, s | 17.0 [16.1–18.2] | 17.3 [16.5–18.1] | 0.212 |
| FIB, g/L | 4.24 [3.13–5.59] | 3.84 [3.08–4.79] | 0.041 |
| D-dimer, mg/L | 1.04 [0.44–2.32] | 0.44 [0.25–0.83] | <0.001 |
| PLR | 109.21 [56.02–164.00] | 107.45 [70.09–161.29] | 0.268 |
| CALLY | 3.77 [1.26–9.28] | 13.98 [4.44–34.90] | <0.001 |
| CLR | 0.09 [0.03–0.24] | 0.32 [0.11–0.77] | <0.001 |
| NLR | 2.43 [1.07–5.47] | 1.63 [0.99–3.31] | 0.002 |
| dNLR | 0.88 [0.81–0.93] | 0.85 [0.81–0.90] | 0.049 |
| SII | 655.9 [296.2–1779.3] | 512.6 [253.5–930.0] | 0.001 |
| NPAR | 15.80 ± 5.13 | 12.89 ± 4.34 | <0.001 |
| MLR | 2.69 [1.30–5.95] | 1.90 [1.21–3.76] | 0.001 |
| SIRI | 2.51 [0.96–6.19] | 1.19 [0.48–2.46] | <0.001 |

Continuous variables are presented as median [Q1–Q3], except HCT, potassium, and NPAR, which are presented as mean ± SD. Q1 and Q3 are the first and third quartiles and delimit the interquartile range (IQR); SD, standard deviation. Normality was assessed within each group using the Shapiro–Wilk test. Variables with P > 0.05 in both groups are shown as mean ± SD; other continuous variables are shown as median [Q1–Q3]. Sex is presented as n (%), and cell percentages are continuous measurements. Normally distributed variables were compared using Welch’s t-test; other continuous variables were compared using the Mann–Whitney U test with continuity correction. Abbreviations are defined preceding Table S1. CALLY = ALB × LYM/CRP and MLR = (MONO + NEU)/LYM, using the completed analysis-file definitions.

## Supplementary Table S2. Clinical baseline characteristics of the sepsis and non-sepsis groups in the external cohort

| **Characteristic** | **Sepsis group (n = 141)** | **Non-sepsis group (n = 211)** | **P value** |
| --- | --- | --- | --- |
| Sex, n (%) | Male, 84 (59.6%) Female, 57 (40.4%) | Male, 124 (58.8%) Female, 87 (41.2%) | 0.880 |
| Age, years | 0.6 [0.2–3.3] | 1.5 [0.3–4.8] | 0.002 |
| WBC, ×10⁹/L | 10.05 [4.17–16.11] | 10.07 [6.89–13.43] | 0.556 |
| Neutrophils (%) | 62.9 [45.2–79.9] | 66.6 [49.2–80.4] | 0.404 |
| Lymphocytes (%) | 28.3 [14.4–47.3] | 24.6 [13.9–43.8] | 0.381 |
| Monocytes (%) | 4.1 [2.5–7.7] | 5.1 [3.2–7.7] | 0.100 |
| Eosinophils (%) | 0.5 [0.1–1.0] | 0.5 [0.1–1.0] | 0.718 |
| Basophils (%) | 0.2 [0.1–0.3] | 0.2 [0.1–0.3] | 0.466 |
| NEU, ×10⁹/L | 5.37 [2.20–11.79] | 6.21 [3.36–9.66] | 0.597 |
| LYM, ×10⁹/L | 1.96 [1.03–3.61] | 2.20 [1.31–3.77] | 0.193 |
| MONO, ×10⁹/L | 0.41 [0.13–0.92] | 0.52 [0.25–0.79] | 0.101 |
| EOS, ×10⁹/L | 0.04 [0.01–0.11] | 0.04 [0.01–0.11] | 0.466 |
| BASO, ×10⁹/L | 0.02 [0.00–0.04] | 0.02 [0.01–0.03] | 0.948 |
| RBC, ×10¹²/L | 3.56 [2.92–3.99] | 4.05 [3.54–4.45] | <0.001 |
| Hb, g/L | 97.0 [83.0–110.0] | 110.0 [97.0–119.0] | <0.001 |
| MCV, fL | 84.8 [79.2–92.4] | 84.9 [80.7–89.7] | 0.871 |
| MCH, pg | 28.0 [25.9–30.2] | 27.6 [26.4–29.4] | 0.252 |
| MCHC, g/L | 329.0 [320.0–340.0] | 326.0 [315.0–334.5] | 0.014 |
| HCT (%) | 29.7 [24.9–33.7] | 33.4 [30.2–37.0] | <0.001 |
| RDW (%) | 14.3 [13.4–15.7] | 13.9 [13.1–15.1] | 0.033 |
| PLT, ×10⁹/L | 188 [75–344] | 320 [222–404] | <0.001 |
| PDW (%) | 15.9 [15.2–16.5] | 15.7 [15.3–16.1] | 0.021 |
| CRP, mg/L | 54.0 [13.0–106.0] | 5.0 [3.0–16.7] | <0.001 |
| PCT, ng/mL | 5.29 [0.36–31.73] | 0.12 [0.06–0.37] | <0.001 |
| K, mmol/L | 3.7 [3.3–4.3] | 3.9 [3.5–4.3] | 0.046 |
| Na, mmol/L | 136 [130–140] | 137 [134–139] | 0.057 |
| Cl, mmol/L | 107 [103–110] | 106 [102–109] | 0.072 |
| Ca, mmol/L | 1.12 [1.02–1.20] | 1.17 [1.10–1.25] | <0.001 |
| P, mmol/L | 1.49 [1.05–1.87] | 1.55 [1.29–1.85] | 0.375 |
| Mg, mmol/L | 0.85 [0.73–0.94] | 0.88 [0.82–0.95] | 0.007 |
| GLU, mmol/L | 5.9 [4.6–7.6] | 6.5 [5.6–8.2] | <0.001 |
| ALT, U/L | 33.0 [16.0–79.0] | 21.0 [15.0–32.5] | <0.001 |
| AST, U/L | 60.0 [35.0–211.0] | 39.0 [29.0–52.0] | <0.001 |
| GGT, U/L | 31.0 [14.0–102.0] | 20.0 [12.0–44.0] | <0.001 |
| ALP, U/L | 145.0 [98.0–231.0] | 184.0 [136.0–265.0] | <0.001 |
| TBIL, µmol/L | 10.1 [6.5–23.3] | 7.1 [4.9–10.8] | <0.001 |
| DBIL, µmol/L | 4.5 [2.2–11.7] | 1.9 [1.1–3.6] | <0.001 |
| IBIL, µmol/L | 6.1 [3.8–12.9] | 4.9 [3.4–7.1] | 0.008 |
| TP, g/L | 54.1 [45.1–60.3] | 63.4 [55.9–69.5] | <0.001 |
| ALB, g/L | 31.11 ± 7.62 | 38.59 ± 5.69 | <0.001 |
| GLOB, g/L | 20.2 [16.2–25.2] | 23.6 [19.2–28.5] | <0.001 |
| A/G | 1.57 [1.20–2.03] | 1.72 [1.33–2.12] | 0.039 |
| BUN, mmol/L | 4.8 [3.0–7.6] | 3.2 [2.4–4.5] | <0.001 |
| CREA, µmol/L | 43.0 [34.2–75.0] | 39.0 [31.0–49.0] | <0.001 |
| UA, µmol/L | 293.0 [212.2–460.0] | 240.0 [170.5–331.0] | <0.001 |
| LDH, U/L | 411.0 [279.0–806.0] | 326.0 [277.0–437.5] | 0.002 |
| CK, U/L | 93.0 [37.0–313.0] | 101.0 [55.5–204.0] | 0.974 |
| CK-MB, U/L | 29 [15–61] | 28 [21–41] | 0.648 |
| TG, mmol/L | 1.19 [0.75–1.83] | 0.92 [0.67–1.29] | <0.001 |
| CHOL, mmol/L | 2.44 [1.78–3.22] | 3.41 [2.78–4.16] | <0.001 |
| PT, s | 11.7 [11.5–12.0] | 11.8 [11.6–12.0] | 0.141 |
| INR | 1.16 [1.01–1.51] | 1.02 [0.94–1.10] | <0.001 |
| APTT, s | 42.9 [34.1–56.2] | 29.6 [25.2–39.7] | <0.001 |
| TT, s | 18.6 [18.2–19.2] | 18.8 [18.4–19.4] | 0.008 |
| FIB, g/L | 1.77 [1.03–3.24] | 2.06 [1.41–2.88] | 0.310 |
| D-dimer, mg/L | 2.52 [1.00–5.25] | 0.78 [0.40–1.73] | <0.001 |
| PLR | 94.74 [30.17–171.05] | 136.14 [76.25–200.75] | <0.001 |
| CALLY | 1.39 [0.38–6.10] | 13.08 [4.18–37.72] | <0.001 |
| CLR | 0.05 [0.01–0.18] | 0.33 [0.12–0.94] | <0.001 |
| NLR | 2.28 [1.00–5.60] | 2.72 [1.13–5.66] | 0.594 |
| dNLR | 0.913 [0.834–0.954] | 0.911 [0.850–0.944] | 0.957 |
| SII | 317.8 [120.8–1268.2] | 728.1 [317.3–1706.4] | <0.001 |
| NPAR | 20.40 [14.21–27.07] | 17.00 [13.23–20.31] | <0.001 |
| MLR | 2.65 [1.13–5.95] | 3.02 [1.26–6.07] | 0.579 |
| SIRI | 1.06 [0.21–3.69] | 1.33 [0.46–2.92] | 0.320 |

Continuous variables are presented as median [Q1–Q3], except albumin, which is presented as mean ± SD. Q1 and Q3 are the first and third quartiles and delimit the interquartile range (IQR); SD, standard deviation. Normality was assessed within each group using the Shapiro–Wilk test. Variables with P > 0.05 in both groups are shown as mean ± SD; other continuous variables are shown as median [Q1–Q3]. Sex is presented as n (%), and cell percentages are continuous measurements. Normally distributed variables were compared using Welch’s t-test; other continuous variables were compared using the Mann–Whitney U test with continuity correction. Abbreviations are defined preceding Table S1. CALLY = ALB × LYM/CRP and MLR = (MONO + NEU)/LYM, using the completed analysis-file definitions.

## Supplementary Table S3. Distributions in the training cohort before and after imputation

| **Characteristic** | **Missing before, n (%)** | **Before imputation** | **After imputation** | **P value** |
| --- | --- | --- | --- | --- |
| WBC, ×10⁹/L | 0 (0.0) | 9.60 [6.54–14.42] | 9.60 [6.54–14.42] | 1.000 |
| Neutrophils (%) | 0 (0.0) | 56.6 [44.8–71.4] | 56.6 [44.8–71.4] | 1.000 |
| Lymphocytes (%) | 0 (0.0) | 32.5 [19.5–44.1] | 32.5 [19.5–44.1] | 1.000 |
| Monocytes (%) | 8 (1.9) | 7.4 [5.6–9.9] | 7.5 [5.6–9.9] | 0.952 |
| Eosinophils (%) | 9 (2.1) | 0.4 [0.1–1.3] | 0.4 [0.1–1.3] | 0.888 |
| Basophils (%) | 9 (2.1) | 0.1 [0.0–0.2] | 0.1 [0.0–0.2] | 0.875 |
| NEU, ×10⁹/L | 1 (0.2) | 5.32 [3.28–8.33] | 5.32 [3.29–8.37] | 0.984 |
| LYM, ×10⁹/L | 1 (0.2) | 2.60 [1.74–4.55] | 2.61 [1.75–4.55] | 0.996 |
| MONO, ×10⁹/L | 5 (1.2) | 0.70 [0.46–1.10] | 0.70 [0.46–1.10] | 0.958 |
| EOS, ×10⁹/L | 8 (1.9) | 0.04 [0.01–0.15] | 0.04 [0.01–0.15] | 0.984 |
| BASO, ×10⁹/L | 9 (2.1) | 0.01 [0.00–0.02] | 0.01 [0.00–0.02] | 0.955 |
| RBC, ×10¹²/L | 4 (0.9) | 4.40 [4.09–4.69] | 4.40 [4.09–4.70] | 0.940 |
| Hb, g/L | 0 (0.0) | 120.5 [112.0–129.0] | 120.5 [112.0–129.0] | 1.000 |
| MCV, fL | 9 (2.1) | 82.1 [78.8–85.1] | 82.1 [78.8–85.1] | 0.970 |
| MCH, pg | 9 (2.1) | 27.7 [26.5–28.7] | 27.7 [26.5–28.7] | 0.974 |
| MCHC, g/L | 9 (2.1) | 337.0 [329.0–344.0] | 337.0 [329.0–344.0] | 0.865 |
| HCT (%) | 9 (2.1) | 35.8 [33.0–38.5] | 35.8 [33.0–38.5] | 0.855 |
| RDW (%) | 9 (2.1) | 13.3 [12.8–14.1] | 13.3 [12.8–14.1] | 0.993 |
| PLT, ×10⁹/L | 2 (0.5) | 282 [225–365] | 282 [226–365] | 0.999 |
| PDW (%) | 9 (2.1) | 15.8 [15.5–16.0] | 15.8 [15.5–16.0] | 0.995 |
| CRP, mg/L | 2 (0.5) | 13.8 [4.2–33.6] | 13.8 [4.2–33.7] | 0.993 |
| PCT, ng/mL | 18 (4.2) | 0.21 [0.10–0.55] | 0.22 [0.10–0.58] | 0.808 |
| K, mmol/L | 1 (0.2) | 4.35 [3.98–4.72] | 4.35 [3.98–4.72] | 0.939 |
| Na, mmol/L | 1 (0.2) | 137 [135–139] | 137 [135–139] | 0.959 |
| Cl, mmol/L | 1 (0.2) | 101.3 [99.0–103.4] | 101.3 [99.0–103.4] | 0.995 |
| Ca, mmol/L | 0 (0.0) | 2.36 [2.25–2.46] | 2.36 [2.25–2.46] | 1.000 |
| P, mmol/L | 1 (0.2) | 1.48 [1.30–1.66] | 1.48 [1.30–1.66] | 0.944 |
| Mg, mmol/L | 1 (0.2) | 0.92 [0.86–0.97] | 0.92 [0.86–0.97] | 0.995 |
| GLU, mmol/L | 1 (0.2) | 5.53 [4.99–6.40] | 5.53 [5.00–6.40] | 0.963 |
| ALT, U/L | 0 (0.0) | 17.0 [12.0–28.0] | 17.0 [12.0–28.0] | 1.000 |
| AST, U/L | 0 (0.0) | 37.1 [28.0–48.6] | 37.1 [28.0–48.6] | 1.000 |
| GGT, U/L | 0 (0.0) | 12.0 [9.0–17.0] | 12.0 [9.0–17.0] | 1.000 |
| ALP, U/L | 0 (0.0) | 186.3 [151.1–237.0] | 186.3 [151.1–237.0] | 1.000 |
| TBIL, µmol/L | 0 (0.0) | 5.5 [3.9–7.6] | 5.5 [3.9–7.6] | 1.000 |
| DBIL, µmol/L | 0 (0.0) | 1.7 [1.1–2.8] | 1.7 [1.1–2.8] | 1.000 |
| IBIL, µmol/L | 0 (0.0) | 3.7 [2.6–5.0] | 3.7 [2.6–5.0] | 1.000 |
| TP, g/L | 0 (0.0) | 66.9 [63.0–70.5] | 66.9 [63.0–70.5] | 1.000 |
| ALB, g/L | 0 (0.0) | 43.1 [40.0–45.7] | 43.1 [40.0–45.7] | 1.000 |
| GLOB, g/L | 0 (0.0) | 23.5 [20.2–26.6] | 23.5 [20.2–26.6] | 1.000 |
| A/G | 1 (0.2) | 1.8 [1.6–2.2] | 1.8 [1.6–2.2] | 0.948 |
| BUN, mmol/L | 3 (0.7) | 3.5 [2.7–4.4] | 3.5 [2.7–4.4] | 0.999 |
| CREA, µmol/L | 2 (0.5) | 29.0 [23.0–36.0] | 29.0 [23.0–36.0] | 0.969 |
| UA, µmol/L | 0 (0.0) | 236.0 [195.0–297.0] | 236.0 [195.0–297.0] | 1.000 |
| LDH, U/L | 2 (0.5) | 288.5 [238.3–353.8] | 288.5 [238.8–354.0] | 0.994 |
| CK, U/L | 1 (0.2) | 89.0 [59.0–138.3] | 89.0 [59.0–138.7] | 0.984 |
| CK-MB, U/L | 1 (0.2) | 27 [20–35] | 27 [20–35] | 1.000 |
| TG, mmol/L | 0 (0.0) | 1.00 [0.71–1.40] | 1.00 [0.71–1.40] | 1.000 |
| CHOL, mmol/L | 1 (0.2) | 3.60 [3.10–4.20] | 3.60 [3.10–4.20] | 0.959 |
| PT, s | 61 (14.1) | 13.5 [12.9–14.2] | 13.5 [12.9–14.2] | 0.939 |
| INR | 61 (14.1) | 1.04 [0.98–1.11] | 1.04 [0.98–1.10] | 0.898 |
| APTT, s | 61 (14.1) | 38.3 [35.3–41.5] | 38.4 [35.3–41.6] | 0.969 |
| TT, s | 61 (14.1) | 17.2 [16.4–18.1] | 17.3 [16.4–18.1] | 0.727 |
| FIB, g/L | 61 (14.1) | 3.95 [3.15–4.88] | 3.92 [3.10–4.87] | 0.801 |
| D-dimer, mg/L | 73 (16.9) | 0.52 [0.30–1.04] | 0.52 [0.26–1.05] | 0.987 |
| PLR | 3 (0.7) | 107.49 [68.47–163.52] | 107.47 [68.33–163.51] | 0.973 |
| CALLY | 3 (0.7) | 8.70 [3.22–29.60] | 8.70 [3.21–29.43] | 0.969 |
| CLR | 3 (0.7) | 0.20 [0.08–0.69] | 0.20 [0.08–0.69] | 0.970 |
| NLR | 1 (0.2) | 1.76 [1.00–3.62] | 1.77 [1.00–3.62] | 1.000 |
| dNLR | 1 (0.2) | 0.86 [0.81–0.91] | 0.86 [0.81–0.91] | 0.993 |
| SII | 3 (0.7) | 542.8 [266.3–1092.0] | 549.1 [266.9–1092.4] | 0.995 |
| NPAR | 0 (0.0) | 13.39 [10.52–16.91] | 13.39 [10.52–16.91] | 1.000 |
| MLR | 5 (1.2) | 2.06 [1.21–4.20] | 2.06 [1.23–4.10] | 0.967 |
| SIRI | 5 (1.2) | 1.32 [0.55–2.91] | 1.32 [0.56–2.93] | 0.981 |

Values are median [Q1–Q3]. Missing percentages use the final cohort size (n = 432). Before imputation, 525 of 24,192 values (2.17%) were missing across 56 source variables. Age and sex were complete and are included in this 56-variable total. The completed dataset contains values for all variables. Two-sided Welch t-tests compare the observed and completed distributions as descriptive checks. NPAR values were identical before and after imputation because NEU% and ALB were complete. CALLY is shown on the completed-CSV scale, ALB × LYM/CRP; MLR = (MONO + NEU)/LYM. Abbreviations are defined preceding Table S1.

## Supplementary Table S4. Distributions in the external cohort before and after imputation

| **Characteristic** | **Missing before, n (%)** | **Before imputation** | **After imputation** | **P value** |
| --- | --- | --- | --- | --- |
| WBC, ×10⁹/L | 0 (0.0) | 10.06 [6.14–14.81] | 10.06 [6.14–14.81] | 1.000 |
| Neutrophils (%) | 0 (0.0) | 65.2 [47.3–80.1] | 65.2 [47.3–80.1] | 1.000 |
| Lymphocytes (%) | 6 (1.7) | 26.0 [14.4–44.4] | 26.0 [14.0–44.5] | 0.977 |
| Monocytes (%) | 3 (0.9) | 4.7 [2.8–7.7] | 4.8 [2.8–7.7] | 0.973 |
| Eosinophils (%) | 1 (0.3) | 0.5 [0.1–1.0] | 0.5 [0.1–1.0] | 0.987 |
| Basophils (%) | 0 (0.0) | 0.2 [0.1–0.3] | 0.2 [0.1–0.3] | 1.000 |
| NEU, ×10⁹/L | 2 (0.6) | 5.87 [2.97–10.34] | 5.91 [3.00–10.41] | 0.729 |
| LYM, ×10⁹/L | 2 (0.6) | 2.12 [1.17–3.62] | 2.12 [1.18–3.69] | 0.755 |
| MONO, ×10⁹/L | 2 (0.6) | 0.48 [0.22–0.82] | 0.48 [0.23–0.82] | 0.775 |
| EOS, ×10⁹/L | 2 (0.6) | 0.04 [0.01–0.11] | 0.04 [0.01–0.11] | 0.978 |
| BASO, ×10⁹/L | 2 (0.6) | 0.02 [0.01–0.03] | 0.02 [0.01–0.03] | 0.754 |
| RBC, ×10¹²/L | 0 (0.0) | 3.83 [3.26–4.32] | 3.83 [3.26–4.32] | 1.000 |
| Hb, g/L | 0 (0.0) | 106.0 [92.0–117.0] | 106.0 [92.0–117.0] | 1.000 |
| MCV, fL | 0 (0.0) | 84.8 [80.0–90.6] | 84.8 [80.0–90.6] | 1.000 |
| MCH, pg | 0 (0.0) | 27.7 [26.3–29.6] | 27.7 [26.3–29.6] | 1.000 |
| MCHC, g/L | 0 (0.0) | 327.0 [316.8–337.0] | 327.0 [316.8–337.0] | 1.000 |
| HCT (%) | 0 (0.0) | 32.10 [28.18–36.20] | 32.10 [28.18–36.20] | 1.000 |
| RDW (%) | 0 (0.0) | 14.0 [13.2–15.4] | 14.0 [13.2–15.4] | 1.000 |
| PLT, ×10⁹/L | 0 (0.0) | 269.0 [155.0–373.3] | 269.0 [155.0–373.3] | 1.000 |
| PDW (%) | 1 (0.3) | 15.7 [15.2–16.3] | 15.7 [15.2–16.3] | 0.960 |
| CRP, mg/L | 42 (11.9) | 9.0 [4.0–33.0] | 11.2 [4.0–48.0] | 0.076 |
| PCT, ng/mL | 32 (9.1) | 0.27 [0.08–1.48] | 0.29 [0.08–1.80] | 0.427 |
| K, mmol/L | 0 (0.0) | 3.80 [3.40–4.30] | 3.80 [3.40–4.30] | 1.000 |
| Na, mmol/L | 0 (0.0) | 137 [133–140] | 137 [133–140] | 1.000 |
| Cl, mmol/L | 0 (0.0) | 106 [102–109] | 106 [102–109] | 1.000 |
| Ca, mmol/L | 0 (0.0) | 1.15 [1.07–1.23] | 1.15 [1.07–1.23] | 1.000 |
| P, mmol/L | 1 (0.3) | 1.54 [1.20–1.86] | 1.54 [1.20–1.86] | 0.982 |
| Mg, mmol/L | 108 (30.7) | 0.87 [0.81–0.95] | 0.87 [0.80–0.95] | 0.634 |
| GLU, mmol/L | 14 (4.0) | 6.3 [5.3–7.9] | 6.3 [5.3–7.9] | 0.982 |
| ALT, U/L | 0 (0.0) | 24.0 [15.0–45.0] | 24.0 [15.0–45.0] | 1.000 |
| AST, U/L | 0 (0.0) | 42.0 [29.8–73.0] | 42.0 [29.8–73.0] | 1.000 |
| GGT, U/L | 0 (0.0) | 24.0 [12.0–55.0] | 24.0 [12.0–55.0] | 1.000 |
| ALP, U/L | 0 (0.0) | 170.0 [119.5–247.5] | 170.0 [119.5–247.5] | 1.000 |
| TBIL, µmol/L | 10 (2.8) | 8.2 [5.2–14.5] | 7.9 [5.3–14.2] | 0.899 |
| DBIL, µmol/L | 2 (0.6) | 2.7 [1.4–5.9] | 2.7 [1.4–5.9] | 0.996 |
| IBIL, µmol/L | 6 (1.7) | 5.0 [3.4–8.8] | 5.1 [3.5–8.6] | 0.964 |
| TP, g/L | 0 (0.0) | 59.1 [52.3–66.9] | 59.1 [52.3–66.9] | 1.000 |
| ALB, g/L | 0 (0.0) | 36.3 [30.8–40.7] | 36.3 [30.8–40.7] | 1.000 |
| GLOB, g/L | 1 (0.3) | 22.1 [18.1–27.1] | 22.1 [18.0–27.0] | 0.951 |
| A/G | 0 (0.0) | 1.64 [1.31–2.09] | 1.64 [1.31–2.09] | 1.000 |
| BUN, mmol/L | 0 (0.0) | 3.5 [2.5–5.7] | 3.5 [2.5–5.7] | 1.000 |
| CREA, µmol/L | 5 (1.4) | 41.0 [31.9–54.3] | 41.0 [32.0–55.1] | 0.917 |
| UA, µmol/L | 0 (0.0) | 259.5 [183.9–376.0] | 259.5 [183.9–376.0] | 1.000 |
| LDH, U/L | 0 (0.0) | 347.0 [279.0–560.0] | 347.0 [279.0–560.0] | 1.000 |
| CK, U/L | 1 (0.3) | 101.0 [49.0–233.5] | 100.5 [48.8–232.3] | 0.994 |
| CK-MB, U/L | 1 (0.3) | 28.0 [18.5–45.5] | 28.0 [18.8–46.0] | 0.998 |
| TG, mmol/L | 1 (0.3) | 1.04 [0.69–1.46] | 1.04 [0.69–1.45] | 0.993 |
| CHOL, mmol/L | 1 (0.3) | 3.11 [2.35–3.85] | 3.11 [2.35–3.85] | 0.971 |
| PT, s | 16 (4.5) | 11.8 [11.6–12.0] | 11.8 [11.6–12.0] | 0.986 |
| INR | 17 (4.8) | 1.04 [0.97–1.20] | 1.04 [0.96–1.20] | 0.945 |
| APTT, s | 23 (6.5) | 34.4 [26.9–45.9] | 34.8 [27.1–47.7] | 0.593 |
| TT, s | 93 (26.4) | 18.8 [18.3–19.3] | 18.8 [18.3–19.3] | 0.752 |
| FIB, g/L | 95 (27.0) | 2.06 [1.31–3.00] | 2.01 [1.28–2.98] | 0.840 |
| D-dimer, mg/L | 104 (29.5) | 1.01 [0.46–2.78] | 1.08 [0.54–3.69] | 0.118 |
| PLR | 2 (0.6) | 118.53 [56.94–188.53] | 118.20 [56.00–187.80] | 0.976 |
| CALLY | 44 (12.5) | 8.27 [1.80–30.03] | 6.64 [1.34–25.21] | 0.521 |
| CLR | 44 (12.5) | 0.22 [0.06–0.72] | 0.18 [0.04–0.63] | 0.548 |
| NLR | 2 (0.6) | 2.59 [1.07–5.67] | 2.59 [1.09–5.65] | 0.989 |
| dNLR | 2 (0.6) | 0.91 [0.84–0.95] | 0.91 [0.84–0.95] | 0.997 |
| SII | 2 (0.6) | 565.69 [211.88–1571.53] | 571.80 [213.57–1559.68] | 0.999 |
| NPAR | 0 (0.0) | 17.72 [13.39–22.49] | 17.72 [13.39–22.49] | 1.000 |
| MLR | 2 (0.6) | 2.81 [1.23–6.07] | 2.81 [1.23–6.02] | 0.989 |
| SIRI | 2 (0.6) | 1.17 [0.31–3.35] | 1.18 [0.31–3.39] | 0.965 |

Values are median [Q1–Q3]. Missing percentages use the final cohort size (n = 352). Before imputation, 594 of 19,712 values (3.01%) were missing across 56 source variables. Age and sex were complete and are included in this 56-variable total. The completed dataset contains values for all variables. Two-sided Welch t-tests compare the observed and completed distributions as descriptive checks. NPAR values were identical before and after imputation because NEU% and ALB were complete. CALLY is shown on the completed-CSV scale, ALB × LYM/CRP; MLR = (MONO + NEU)/LYM. Abbreviations are defined preceding Table S1.

## Supplementary Table S5. Discriminative performance of candidate clinical features in the training cohort

| **Characteristic** | **AUC (95% CI)** |
| --- | --- |
| Sex | 0.434 (0.383–0.485) |
| Age, years | 0.639 (0.577–0.700) |
| WBC, ×10⁹/L | 0.707 (0.649–0.766) |
| Neutrophils (%) | 0.599 (0.534–0.663) |
| Lymphocytes (%) | 0.602 (0.539–0.666) |
| Monocytes (%) | 0.505 (0.442–0.567) |
| Eosinophils (%) | 0.580 (0.520–0.639) |
| Basophils (%) | 0.620 (0.560–0.680) |
| Neutrophil count, ×10⁹/L | 0.706 (0.649–0.762) |
| Lymphocyte count, ×10⁹/L | 0.544 (0.478–0.610) |
| Monocyte count, ×10⁹/L | 0.683 (0.623–0.742) |
| Eosinophil count, ×10⁹/L | 0.523 (0.462–0.584) |
| Basophil count, ×10⁹/L | 0.538 (0.476–0.601) |
| RBC, ×10¹²/L | 0.662 (0.600–0.723) |
| Hb, g/L | 0.695 (0.637–0.753) |
| MCV, fL | 0.541 (0.474–0.609) |
| MCH, pg | 0.513 (0.444–0.581) |
| MCHC, g/L | 0.552 (0.486–0.617) |
| HCT, % | 0.679 (0.618–0.740) |
| RDW, % | 0.653 (0.590–0.716) |
| PLT, ×10⁹/L | 0.533 (0.469–0.597) |
| PDW, % | 0.453 (0.385–0.520) |
| CRP, mg/L | 0.762 (0.706–0.818) |
| PCT, ng/mL | 0.785 (0.735–0.834) |
| K, mmol/L | 0.614 (0.551–0.678) |
| Na, mmol/L | 0.630 (0.568–0.692) |
| Cl, mmol/L | 0.497 (0.432–0.562) |
| Ca, mmol/L | 0.544 (0.477–0.610) |
| Phosphorus, mmol/L | 0.486 (0.421–0.551) |
| Mg, mmol/L | 0.548 (0.482–0.613) |
| GLU, mmol/L | 0.498 (0.433–0.562) |
| ALT, U/L | 0.677 (0.616–0.738) |
| AST, U/L | 0.526 (0.457–0.595) |
| GGT, U/L | 0.682 (0.621–0.744) |
| ALP, U/L | 0.554 (0.492–0.616) |
| TBIL, µmol/L | 0.637 (0.573–0.701) |
| DBIL, µmol/L | 0.673 (0.612–0.733) |
| IBIL, µmol/L | 0.558 (0.491–0.625) |
| TP, g/L | 0.705 (0.645–0.765) |
| ALB, g/L | 0.712 (0.653–0.770) |
| GLOB, g/L | 0.622 (0.556–0.689) |
| A/G ratio | 0.536 (0.467–0.604) |
| BUN, mmol/L | 0.515 (0.449–0.581) |
| CREA, µmol/L | 0.608 (0.546–0.670) |
| UA, µmol/L | 0.637 (0.574–0.699) |
| LDH, U/L | 0.550 (0.484–0.616) |
| CK, U/L | 0.623 (0.559–0.688) |
| CK-MB, U/L | 0.534 (0.470–0.598) |
| TG, mmol/L | 0.581 (0.520–0.641) |
| CHOL, mmol/L | 0.610 (0.543–0.677) |
| PT, s | 0.603 (0.538–0.668) |
| INR | 0.575 (0.510–0.640) |
| APTT, s | 0.531 (0.464–0.597) |
| TT, s | 0.539 (0.472–0.607) |
| FIB, g/L | 0.565 (0.499–0.631) |
| D-dimer, mg/L | 0.690 (0.627–0.753) |
| PLR | 0.465 (0.399–0.531) |
| CALLY | 0.712 (0.653–0.771) |
| CLR | 0.702 (0.642–0.762) |
| NLR | 0.599 (0.535–0.663) |
| dNLR | 0.562 (0.498–0.627) |
| SII | 0.603 (0.539–0.667) |
| NPAR | 0.659 (0.596–0.722) |
| MLR | 0.602 (0.538–0.665) |
| SIRI | 0.681 (0.621–0.740) |

Sex was included as a binary variable. AUCs and DeLong 95% CIs were calculated from the final training dataset (n = 432), using the original code’s automatic ROC direction. NPAR summaries use the formula defined in Section 2.2. Figure 2 presents the selection results used for the 12-predictor models; this table presents the supplementary univariable summaries. AUC, area under the receiver operating characteristic curve; CI, confidence interval. Other abbreviations are defined preceding Table S1.

## Supplementary Table S6. Apparent performance of the exploratory Gram-stain classification model

| **Accuracy** | **Sensitivity** | **Specificity** | **Precision** | **F1 score** | **AUC (95% CI)** |
| --- | --- | --- | --- | --- | --- |
| 0.717 | 0.571 | 0.803 | 0.632 | 0.600 | 0.753 (0.661–0.844) |

Values are proportions unless otherwise indicated. Gram-positive infection is the positive class. At a classification threshold of 0.44, Figure S1C contains 24 true positives, 14 false positives, 18 false negatives, and 57 true negatives. Accuracy = 81/113 = 0.717; sensitivity = 24/42 = 0.571; specificity = 57/71 = 0.803; precision = 24/38 = 0.632; F1 score = 48/80 = 0.600. The dashed line at 0.5 belongs to the probability-distribution panel (S1B). AUC, area under the receiver operating characteristic curve; CI, confidence interval. These are apparent performance estimates from the development sub-cohort.
